# A high aspect ratio surface micromachined accelerometer based on a SiC-CNT composite material

**DOI:** 10.1038/s41378-024-00672-x

**Published:** 2024-03-22

**Authors:** Jiarui Mo, Shreyas Shankar, Roberto Pezone, Guoqi Zhang, Sten Vollebregt

**Affiliations:** Laboratory of Electronic Components, Technology and Materials (ECTM), Department of Microelectronics, Delft University of Technology, Delft, The Netherlands

**Keywords:** Nanoscale materials, Electrical and electronic engineering

## Abstract

Silicon carbide (SiC) is recognized as an excellent material for microelectromechanical systems (MEMS), especially those operating in challenging environments, such as high temperature, high radiation, and corrosive environments. However, SiC bulk micromachining is still a challenge, which hinders the development of complex SiC MEMS. To address this problem, we present the use of a carbon nanotube (CNT) array coated with amorphous SiC (a-SiC) as an alternative composite material to enable high aspect ratio (HAR) surface micromachining. By using a prepatterned catalyst layer, a HAR CNT array can be grown as a structural template and then densified by uniformly filling the CNT bundle with LPCVD a-SiC. The electrical properties of the resulting SiC-CNT composite were characterized, and the results indicated that the electrical resistivity was dominated by the CNTs. To demonstrate the use of this composite in MEMS applications, a capacitive accelerometer was designed, fabricated, and measured. The fabrication results showed that the composite is fully compatible with the manufacturing of surface micromachining devices. The Young’s modulus of the composite was extracted from the measured spring constant, and the results show a great improvement in the mechanical properties of the CNTs after coating with a-SiC. The accelerometer was electrically characterized, and its functionality was confirmed using a mechanical shaker.

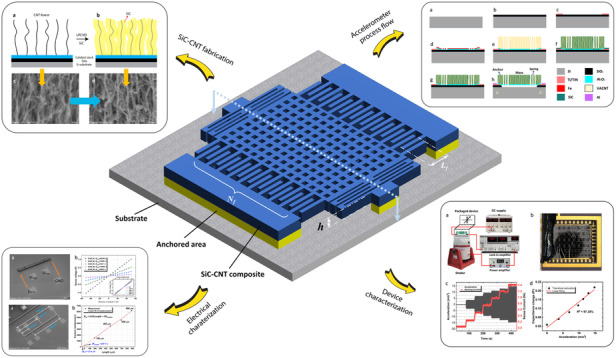

## Introduction

The demand for microelectromechanical systems (MEMS) that are compatible with harsh environments is increasing^1^. Traditional silicon (Si)-based MEMS cannot survive in extreme environments, such as high temperatures, because Si junction-based devices suffer from significant leakage at high temperatures. Additionally, Si experiences plastic deformation at 500 °C^1^, which is fatal for devices with movable components. Silicon carbide (SiC), a representative wide bandgap material, has been recognized as a promising candidate for MEMS devices that require operation in harsh environments^2–6^. Due to SiC’s wide bandgap, SiC-based electronics can work at a much higher temperature than their silicon counterparts. SiC has a critical electric field higher than that of Si and a thermal conductivity approximately three times greater than that of Si. In terms of mechanical properties, SiC has a Young’s modulus 2.4 to 4.3 times greater (depending on the polytype) than silicon, and it is one of the hardest materials ever discovered^7^. Additionally, SiC retains its mechanical properties even at very high temperatures.

Despite these advantages, the development of SiC MEMS is impeded by the lack of sufficient bulk micromachining processes. The mechanical stability and chemical inertness of SiC decrease the effectiveness of conventional bulk etching methods. SiC is stable in most known aqueous etching solutions. The exceptions are phosphoric acid and alkaline solutions of K_3_Fe(CN)_6_. However, the aqueous etching processes exhibit issues such as a low etch rate and oxidation^8^. Molten salts, especially KOH, can etch SiC and have been widely used for studying defects in SiC, but etching requires a high temperature and a dedicated container. Additionally, the process is isotropic and therefore cannot be used for fabricating high aspect ratio (HAR) structures^9^. Reactive ion etching (RIE) is the most promising technique for SiC bulk micromachining. However, most reported SiC dry etching processes still demonstrate insufficient etching rates (typically 1 *µ*m/min or less), poor selectivity to mask materials, rough etch surfaces, non-vertical profiles, and microtrenching effects^10–13^. As summarized by Dowling et al., the aspect ratio of SiC structures fabricated by plasma etching reported in the literature is usually less than 10, and these structures typically exhibit a “V” shape due to the non-vertical nature of the sidewall^11^.

Surface micromachining is also essential in MEMS fabrication. Mono-crystalline SiC is nearly impossible for this application, as it cannot be grown on sacrificial materials. Fortunately, polycrystalline 3C-SiC can be deposited using chemical vapor deposition (CVD) over an insulating substrate such as silicon dioxide (SiO_2_)^2,4^. However, the SiC layer thickness is generally limited by the low deposition rate and layer stress. For this reason, the poly-SiC device layers in all the reported SiC surface micromachining MEMS devices do not exceed 10 *µ*m^3,5,6,14–17^. This limits several SiC MEMS implementations.

To overcome this limitation, we present the use of a carbon nanotube array reinforced with SiC to form a SiC-CNT composite. The CVD of a vertically aligned carbon nanotube (VACNT) array enables the fast fabrication of HAR structures. The growth rate of CNTs can reach a few tens or hundreds of nanometers per second, and CNTs can reach several millimeters in length with an excellent vertical sidewall^18–20^. However, CNT arrays have foam-like properties because interwoven CNTs are only weakly bonded to each other by van der Waals forces. This makes the CNT array vulnerable to loads from both the vertical and horizontal directions. Fortunately, the porous nature of the CNT array allows us to densify it by filling with other materials, thus producing a reinforced nanocomposite.

In recent years, there has been some research on modifying the intrinsic VACNT properties with different filler materials. Ci et al. created reinforced CNT arrays by infiltrating them with liquid-state polydimethylsiloxane (PDMS). The work showed that the longitudinal modulus increased 33 times, and the damping capability increased 21 times^19^. In 2010, Hutchison et al. showed CNT arrays infiltrated by poly-Si and silicon nitride (Si_3_N_4_). They found that the filler material had improved mechanical properties and that the electrical conductivity was dominated by the CNTs^20^. With this technique, a variety of actuators were implemented as demonstrators. In 2014, Poelma et al. studied the mechanical properties of VACNT arrays coated with low-pressure chemical vapor deposition (LPCVD) amorphous SiC (a-SiC) by nanoindentation. The compressive strength and Young’s modulus of the coated VACNTs significantly increased compared to those of the uncoated CNT array. The authors of that study suggested that the SiC-CNT composite can be useful in applications such as vertical interconnects and 3D supercapacitors^21^. These representative works prove that filler-reinforced CNT arrays have enhanced mechanical properties and show potential for manufacturing MEMS devices.

In this work, we demonstrate the fabrication of a SiC-CNT composite. The electrical properties of this material are studied by characterizing the bulk resistivity and contact resistance with the metal layer. To demonstrate its further use in manufacturing HAR MEMS, we design, fabricate, and characterize a MEMS accelerometer based on this composite. Based on the capacitive measurement result of the accelerometer, Young’s modulus of the SiC-CNT composite is determined. The Young’s modulus of the CNTs increases by 3 orders of magnitude after they are filled with a-SiC. To our knowledge, this accelerometer is the first MEMS sensor that has been fabricated using a CNT array reinforced by a filler.

## Materials and methods

**Fig. 1:**
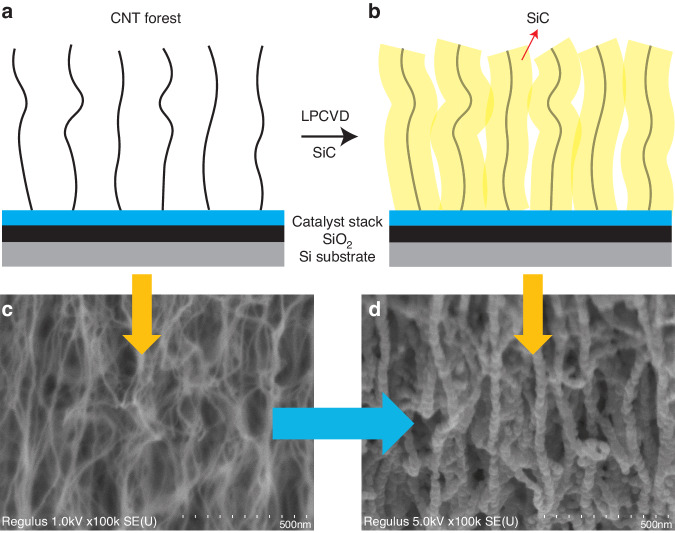
The basic concept of fabricating SiC-CNT composites. **a** VACNT array is grown on a prepatterned catalyst layer (Fe on Al_2_O_3_), **b** LPCVD a-SiC fills the porous CNT array; and **c** the surface of the CNT array before and **d** after being filled by 14 nm a-SiC

**Fig. 2:**
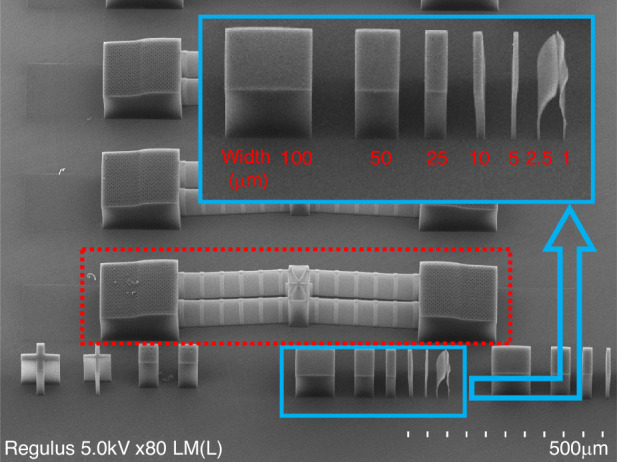
SEM image of the as-grown VACNT array (tilted view). The inset in the blue box shows the unclamped structure, which bends when the HAR reaches 10. The clamped structure in the red box does not bend even though the aspect ratio reaches 20

The concept of our SiC-CNT composites is depicted in Fig. 1. First, a SiO_2_ layer is deposited on the silicon substrate as a diffusion barrier for the catalyst stack^22^. This oxide layer is also used as a sacrificial layer for surface micromachined devices. Then, a catalyst stack for CNT growth is grown by electron beam evaporation. The catalyst stack consists of 20 nm aluminum oxide (Al_2_O_3_) and 2 nm iron (Fe), where the Al_2_O_3_ layer is evaporated prior to Fe to enhance the nucleation density of CNTs from Fe particles^23^. After evaporation, the CNT arrays are grown by CVD using an Aixtron Blackmagic. The deposition procedure consists of an initial activation step in a H_2_ environment at 500 °C for 3 min, followed by an actual growth step with a gas mixture of H_2_/C_2_H_2_ (700/50 sccm) at 80 mbar and 600 °C. The height of the CNT array with respect to the deposition time is summarized in Appendix A Table A1. The deposition rate is tens of micrometers per minute; however, this rate decreases over time. This decrease occurs due to the depletion of the catalyst layer, i.e., Fe in this case, which eventually terminates CNT growth^24^. The cross-section after CNT growth is given in Fig. 1a, and Fig. 1c shows the surface of the as-grown VACNT array, where CNT fibers are weakly interwoven together by van der Waals forces. The highest CNT array was obtained by a 5-min deposition and had a height of 96.3 *µ*m, as shown in Fig. 2. The test structures shown in the inset have aspect ratios ranging from 0.96 to 96.3. Structures with an aspect ratio larger than 10 showed different extents of bending. Notably, the structures that are encircled by the red dashed box in Fig. 2 did not show bending even when the aspect ratio was approximately 20. This occurs because these structures are clamped at both sides to anchors, which act as mechanical supports. It implies that an aspect ratio higher than 20 is still possible if the CNT arrays are properly designed. After the growth of the CNT arrays, the VACNT array was filled with a-SiC, as illustrated in Fig. 1b. The filling is performed by LPCVD of a-SiC. The low deposition rate of a-SiC enhances the infiltration process of the filler into the CNT array, thus producing a denser composite. The deposition used a dichlorobenzene (SiH_2_Cl_2_) and acetylene (C_2_H_2_) gas mixture diluted in hydrogen (H_2_) at 760 °C and at 1 mbar. The flow rates of SiH_2_Cl_2_ and C_2_H_2_ were 3.25 sccm and 21.75 sccm, respectively. This recipe yielded a deposition rate of approximately 0.25 nm/min. The result after deposition is shown in Fig. 1d. Comparing Fig. 1c and 1d, it can be seen that the individual CNT fibers became much thicker after deposition. These coating results are similar to what was reported by Poelma et al.^21^. To verify whether the CNTs deep inside the bulk were also uniformly coated, some structures were cut deliberately with tweezers. As shown in Fig. A1, the amount of a-SiC coated in the bulk is the same as that on the surface. As each individual CNT is coated with a-SiC, it is inferred that the SiC-CNT composite will be as chemically stable as the pure a-SiC which demonstrated excellent inertness to wet chemicals^25,26^. This suggests that SiC-CNT composites have the potential to be applied in corrosive environments. Additionally, the SiC-CNT composite becomes denser with increasing a-SiC deposition time, as shown in Fig. A2. This means that the mechanical properties of the composite can be tuned by conducting different extents of the a-SiC filling process.

### Bulk resistivity

**Fig. 3:**
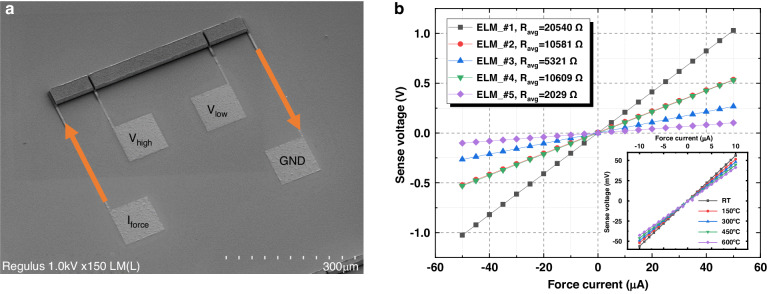
The bulk resistivity measurement of SiC-CNT composite. **a** ELM test structure fabricated with the SiC-CNT composite. **b** Four-probe measurement results for different ELM structures with the dimensions listed in Table 1. Inset: Resistance measured at elevated temperatures

The bulk resistivity of the SiC-CNT composite was characterized by electrical line-width measurements (ELM). The measurements were performed with the four-probe method to eliminate resistance from contacts and probe wires. The ELM structures have an effective length of 300 *µ*m and a height of 10 *µ*m. The widths of the test structures are 10 *µ*m, 20 *µ*m, and 40 *µ*m. A current was forced into the ELM structures, sweeping from −50 *µ*A to +50 *µ*A. The *I*–*V* characteristics are plotted in Fig. 3b. As seen from the measurements, the resistance of the ELM structure scales with the dimension. This result indicates good uniformity of the CNT arrays.

The bulk resistivity is extracted from the measurement results and is listed in Table 1. The bulk resistivities of the coated arrays are very close to each other (0.701 ± 0.010 Ω cm). A sample (Sample #5) without SiC infiltration was also measured and showed an intrinsic resistivity of 0.223 Ω cm. The resistivity of the composite was more than 3 times greater than that of the intrinsic CNT array. The degradation may be attributed to part of the CNT conductive paths being disconnected during a-SiC deposition. Another factor could be the degradation of CNTs during the furnace boat-in process when they are exposed to oxygen and could oxidize. However, compared to the resistivity of a-SiC (on the order of MΩ cm^5^), the bulk resistivity decreases on the order of 10^7^. Therefore, we conclude that the bulk resistivity is dominated by the CNTs. If the resistivity of SiC-CNT composite is compared to that of doped bulk SiC, it would correspond to a doping concentration of ~10^16^ atom/cm^3^ for nitrogen-doped SiC and ~5 × 10^19^ atom/cm^3^ for aluminum-doped SiC^7^.

**Table 1:** Resistance measurements of SiC-coated CNTs

Sample (#)	CNT height (*µ*m)	Width (*µ*m)	Resistance (Ω)	Resistivity (Ω m)
1	10	10	20540	0.00684
2	10	20	10580	0.00705
3	10	40	5321	0.00709
4	20	10	10610	0.00707
5^a^	33	10	2030	0.00223
150 °C	10	40	5155	0.00652
300 °C	10	40	4835	0.00611
450 °C	10	40	4475	0.00566
600 °C	10	40	4135	0.00523

^a^This sample is not coated with a-SiC

The bulk resistivity of the SiC-CNT composite at high temperatures is also studied and is summarized in Table 1. The resistance of the EML structure was measured up to 600 °C. As shown in the inset of Fig. 3b, the SiC-CNT composite still exhibited a linear *I*–*V* relationship at all the measured temperature points, i.e. 150 °C, 300 °C, 450 °C, and 600 °C. In addition, a negative temperature coefficient of resistivity (TCR) of approximately −315 ppm/K is observed. This result aligns well with the TCR of CNTs reported in^27,28^. The negative TCR might be related to the increase in conduction channels in the CNTs with increasing temperature^29^. This further proves that CNTs play a major role in the electrical properties of the composite.

### Contact resistivity

**Fig. 4:**
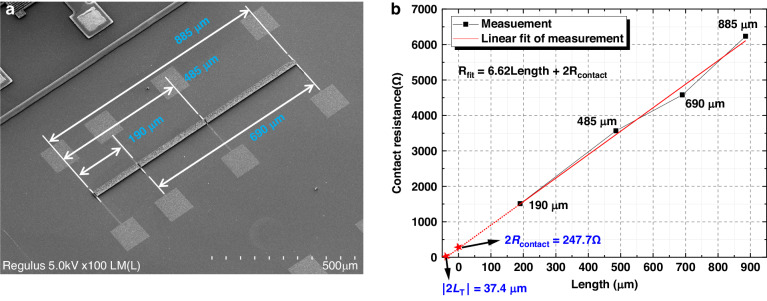
The measurement of specific contact resistivity between the interconnect layer and composite. **a** The TLM structure used in the measurement. **b** Measured resistances from different segments of the TLM structure. The intersection between the curve and the *y*-axis is the value of the contact resistance

To interface with measurement instruments, a conductive interconnection layer is required for wire bonding. Typically, wire bonding should be performed on a surface with low surface roughness to ensure the bondability and reliability of the wire bonds. However, the top surface of the SiC-CNT composite is rough and is not an ideal surface for wire bonding. Therefore it was decided to fabricate the interconnection layer before fabricating the SiC-CNT composite. This means that the interconnection layer needs to withstand the high temperature brought by CNT growth and SiC deposition. Titanium nitride (TiN) was chosen for this purpose because of its high endurance under high-temperature conditions. As a result, the contact region consists of TiN, the SiC-CNT composite, and possibly the Al_2_O_3_/Fe catalyst stack. In principle, Al_2_O_3_ is an insulator and prevents conduction between TiN and CNTs unless the layer is sufficiently thin or if interdiffusion occurs^30^. The contact resistivity of such a contact stack was measured with the transmission line method (TLM). As indicated in Fig. 4a, four resistors with different evaluation lengths, i.e., 190, 485, 690, and 885 *µ*m, were measured. Again, the four-probe method was used for TLM measurements. Figure 4b shows the measured result and linear fitted curve. By extrapolating the fitting curve to the *y*-axis, the sum of the contact resistances on both contacts (2*R*_contact_) is estimated to be 248 Ω. Combining this information with the transfer length (*L*_*T*_) obtained from the *x*-axis intersect, i.e., *L*_*T*_ = 18.7 *µ*m, the specific contact resistivity is found to be 4.63 × 10^*−*4^ Ω cm^2^. Due to the non-conductive nature of Al_2_O_3_, we infer that the conduction could be mostly attributed to electron tunneling between the interface.

### Comb-type capacitive accelerometer

To demonstrate the potential of using SiC-CNT composites for HAR devices, we designed, fabricated, and characterized a classical comb-type capacitive accelerometer fabricated with the SiC-CNT composite. The HAR structure is particularly useful for capacitive sensors because it can effectively increase the surface area that is used for capacitive detection^20^.

**Fig. 5:**
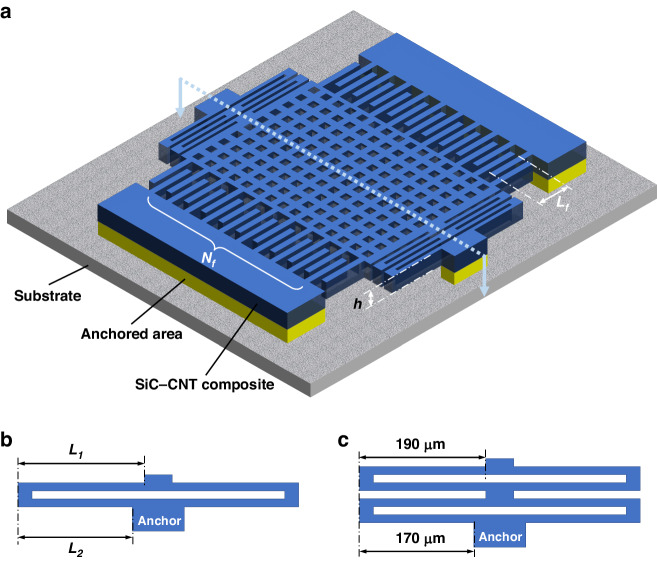
A schematic of the accelerometer. **a** Typical structure of a MEMS comb-type accelerometer, **b** single-meandered spring, **c** double-meandered spring

A schematic of the comb structure capacitive accelerometer is shown in Fig. 5a. The device comprises a proof mass, interdigitated fingers (fixed fingers are anchored to the substrate, and movable fingers are attached to the proof mass), fixed anchors, and folded beams as the spring that connects the proof mass and anchors. Except for the anchors, all the structures are suspended by the surface micromachining process. An array of 4 *µ*m × 4 *µ*m holes with a pitch size of 9 *µ*m was designed on the proof mass to help with releasing the structure. The additional benefit of these release holes is that they act as vapor access holes to enhance the infiltration of a-SiC filler into the CNT array^20^.

## Results and discussion

### Analytical model for an accelerometer

The accelerometer capacitance as a function of the acceleration *C*(*a*) is expressed as follows:

1 $$C\left(a\right)=\frac{\varepsilon {N}_{f}{L}_{f}h}{{d}_{0}-({Ma}/2k)}\approx {C}_{0}\left(1+\frac{{Ma}}{2{d}_{0}k}\right){{\rm{where}}}\,{C}_{0}=\frac{\varepsilon {N}_{f}{L}_{f}h}{{d}_{0}}$$

and the sensitivity to acceleration (*S*) is

2 $$S=\frac{{C}_{o}M}{{2d}_{0}k}$$

where *ε* is the permittivity of the media, *N*_*f*_ is the number of fingers, *L*_*f*_ is the overlap length between the fixed fingers and movable fingers, *h* is the thickness of the SiC-CNT composite, *d*_0_ is the initial distance between two fingers, *C*_0_ is the nominal capacitance without acceleration, *M* is the mass of the proof mass, and *k* is the stiffness of the folded spring structure. To obtain better sensitivity, a higher *C*_0_ is desired. To realize this design goal, *N*_*f*_, *L*_*f*_, and *h* should be kept as high as possible, while *d*_0_ should be minimized. Considering the footprint of the device, *N*_*f*_ and *L*_*f*_ are decided to be 26 and 105 *µ*m, respectively. The height of the SiC-CNT array is 10 *µ*m, resulting in an aspect ratio of approximately 3 to minimize the shadowing effect and to produce mechanically robust structures. Here, as a proof-of-concept design, the geometry tends to be conservative; thus, the aspect ratio is not designed to be the highest that we can achieve. *d*_0_ is kept at 3 *µ*m because individual CNTs in the current growth recipe sometimes protrude from the CNT bundle, which will cause a short circuit between the plates if *d*_0_ is too small. Based on the above device dimensions, *C*_0_ is approximately 80.5 fF, and the mass of the shuttle *M* can be estimated to be 9.06 ng.

As per Eq. (2), the spring constant *k* also plays an important role in the accelerometer sensitivity. Figure 5b shows a typical folded spring, with two segments of length *L*_1_ and *L*_2_ on each side. From^31^, *k* of such a structure can be expressed as:

3 $$k=\left(\frac{{\pi }^{4}}{6}\right)\frac{E{W}^{3}h}{{(2{L}_{1})}^{3}+{(2{L}_{2})}^{3}}$$

where *W* is the width of the beam. In the actual design, two folded springs are cascaded together for a smaller *k*, as shown in Fig. 5c. The first spring has *L*_1_ = 190 *µ*m and *L*_2_ = 170 *µ*m, and for the second spring, *L*_1_ = *L*_2_ = 190 *µ*m. The effective *k* of the cascaded spring is approximately 16.81 N/m.

According to the estimated *C*_0_, *M*, *k*, and the process parameter *d*_0_, the sensitivity of the designed accelerometer is calculated to be 0.07 fF/g. The mechanical properties of the composite, i.e., density and Young’s modulus, are assumed to be determined only by the filling material during the estimation of *M* and *k*.

### Accelerometer fabrication

**Fig. 6:**
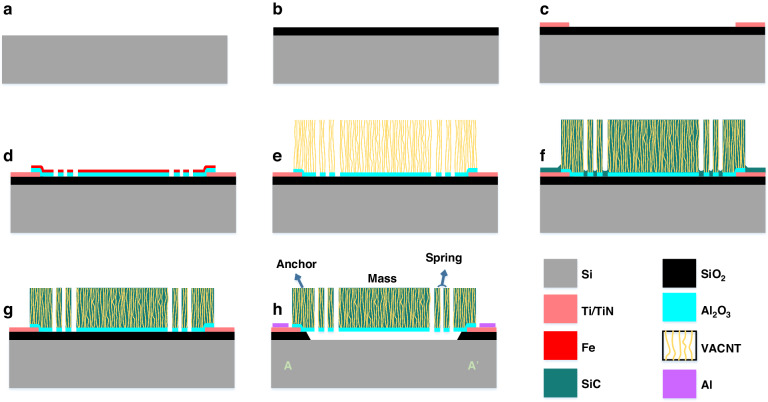
Process overview of the SiC-CNT composite fabrication method. **a** A bare Si substrate, **b** 3000 nm PECVD SiO_2_ deposited on the Si wafer, **c** a Ti/TiN layer sputtered and patterned as electrodes, **d** a catalyst stack (Al_2_O_3_/Fe) fabricated via the liftoff process, **e** a CNT array grown on the prepatterned catalyst, **f** a porous CNT template filled with LPCVD a-SiC, **g** dry etching performed on the entire sample to expose the contact area and the sacrificial layer, **h** a sample metallized by aluminum, followed by VHF to release suspended structures

The fabrication steps of the SiC-CNT accelerometer are summarized in Fig. 6. The process starts with a bare 100 mm silicon wafer (Fig. 6a). A 3000 nm SiO_2_ layer is deposited on the substrate not only as a diffusion barrier but also as a sacrificial layer to enable the fabrication of a surface micromachined device (Fig. 6b). A bilayer consisting of 10 nm Ti (titanium) and 50 nm TiN was then deposited by reactive sputtering. Ti (10 nm) was sputtered prior to the TiN layer to enhance adhesion. The Ti/TiN stack is etched by dichlorine (Cl_2_) and hydrogen bromide (HBr) at 25 °C to form electrical contacts, resulting in the cross-sections shown in Fig. 6c.

The substrate is patterned with negative-tone photoresist, where the mass, spring, fingers, and anchor areas are left open for evaporation. Then, the catalyst stack for CNT growth, i.e., Al_2_O_3_ and Fe, was evaporated as described previously in section 2. After evaporation, the unwanted area of the catalyst material was removed by *N*-methyl pyrrolidone (NMP) at 70 °C with the aid of an ultrasonic bath (Fig. 6d). Using an Aixtron Blackmagic CVD system, a VACNT array with a height of approximately 10 *µ*m was grown on the prepatterned catalyst produced by the lift-off process, as shown in Fig. 6e. The height of the CNT arrays has some non-uniformity across the wafer. From the edge to the center of the 4-inch wafer, the CNT height varies from 9.6 *µ*m to 11.2 *µ*m. This variance mainly occurs due to the non-uniform temperature distribution resulting from the single-zone heating design of the CVD tool. After CNT growth, a thick layer of a-SiC (90 nm) was deposited by LPCVD to fill the VACNT array (Fig. 6f). The thickness of the a-SiC coating was measured with a Si dummy wafer (the same deposition as the process wafer), and the actual thickness was 89.25 nm, with a standard deviation of 0.68 nm. A thick a-SiC layer is used to minimize the voids between the CNTs and to achieve mechanically robust suspended structures. As shown in Fig. 6f, a-SiC deposition not only infiltrates CNTs but also deposits SiC on the entire wafer, thus forming a floor layer that covers the sacrificial oxide and TiN contact pads. This floor layer prevents the sacrificial layer from releasing by vapor hydrogen fluoride (VHF). Additionally, the metal pads cannot have proper connections with instruments with a non-conductive a-SiC on top. Hence, a blanket dry etching step is performed to remove the thin a-SiC layer on the entire wafer, i.e., no photoresist is used to pattern the wafer. Due to the poor selectivity of dry etching, a low-power dry etching recipe was used to avoid over-etching of the TiN pads and excessive etching of the CNT arrays. The etching was carried out for 90 s by a Sentech Etchlab 200 plasma etcher using 13.5 sccm sulfur hexafluoride (SF_6_) and 3.5 sccm oxygen (O_2_) at a pressure of 8 *µ*bar and an RF power of 50 W. This recipe removes most of the covering a-SiC without significantly affecting the height of the array (Fig. 6g).

Aluminum metallization was applied prior to the VHF release of the structure. An aluminum metallization layer was used on top of the TiN pads because we found that the TiN layer was corroded during VHF release. The final step is to release the structure by VHF. A slow etching recipe was used to create a controllable etching process. The mixture consisted of 310 sccm HF, 350 sccm ethanol and 1250 sccm of nitrogen (N_2_) was used, which yielded a SiO_2_ etching rate of 470 nm/min. A schematic cross-section of the final device is shown in Fig. 6h.

**Fig. 7:**
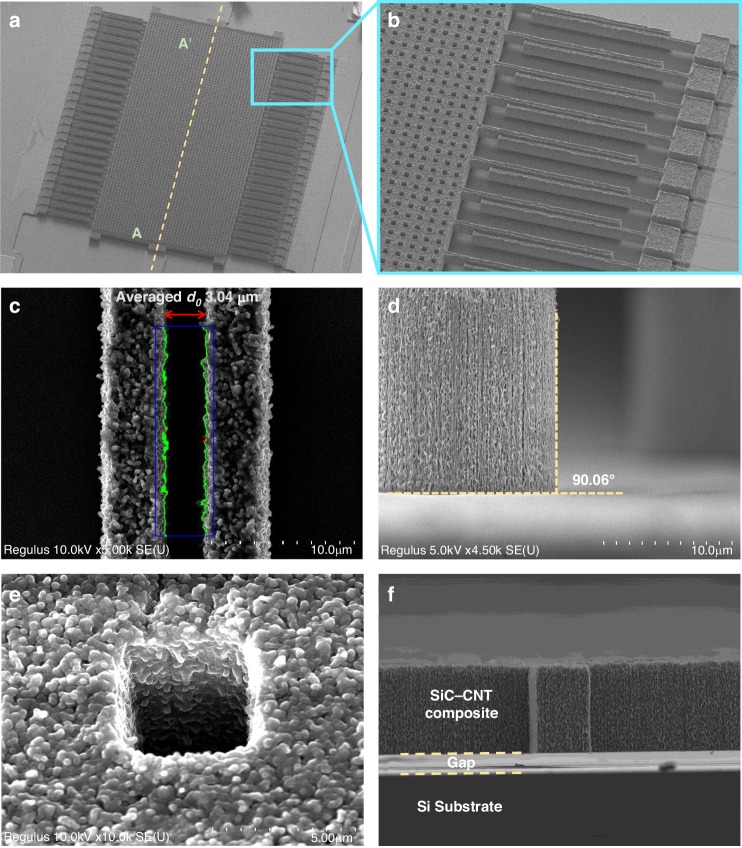
SEM inspection of the fabricated device. **a** A full view of the accelerometer fabricated from the SiC-CNT composite (AA’ cross section corresponds to Fig. 6h). **b** Array of interdigitated fingers attached to the mass. **c** A zoomed-in view of a pair of fingers with the average gap distance indicated, and the green line indicates the feature edges. **d** The sidewall profile of the SiC-CNT composite. **e** A 4 × 4 *µ*m^2^ releasing hole on the mass. **f** A side view of the suspended structure, where the gap can be clearly identified. No obvious out-of-plane deflection can be observed

The completed device was inspected via SEM, and a full view of the device is shown in Fig. 7a. Figure 7b shows the array of the interdigitated fingers. A close-up view of the finger is shown in Fig. 7c, where no physical contact can be found between the movable and static capacitor plates. The gap distance between the fingers *d*_0_ is crucial for determining the base capacitance of the device, so the gap distances of 30 pairs of fingers were measured. The average gap distance is 3.04 *µ*m, which is very close to the design value of *d*_0_ (3 *µ*m). The existing manufacturing error may be attributed to the lithography step, including undesired effects such as overexposure. The green line in 7c indicates the feature edge, and the edge line roughness is calculated to be 297 nm. In Fig. 7d, the sidewall profile of the SiC-CNT composite shows a nearly 90° angle due to the vertical growth of the CNT array. In Fig. 7e, one of the 4 × 4 *µ*m^2^ release holes is shown, which allows a mild VHF recipe to release the structure. Figure 7f shows a 90° tilted view of the suspended part of the device, where no sagging can be observed. These findings demonstrated that the composite can fabricate a stiff enough structure for surface micromachined devices.

### Accelerometer characterization

**Fig. 8:**
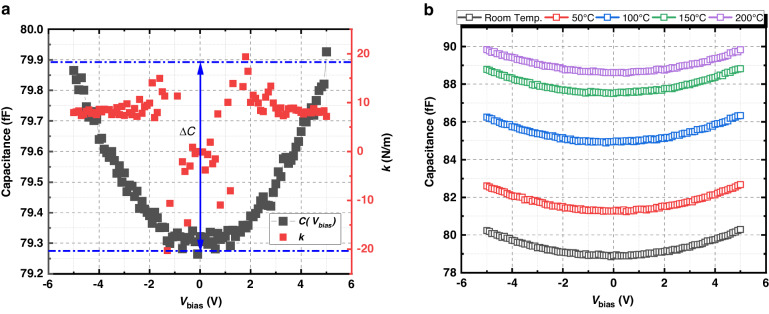
Capacitance measurement on the accelerometer. **a** Measured capacitance at the rest position and the extracted *k* when −5 V *≤ V*_bias_ ≤ 5 V. **b** Measured rest-capacitance at different temperatures

After fabrication, the wafers were diced and characterized at the die level. The capacitances of the accelerometers were measured with an Agilent 4294 A Precision Impedance Analyzer. For each measurement point, a DC voltage bias *V*_*bias*_ is applied to the capacitor, sweeping from −5 V to +5 V with a step of 100 mV. The measured *C*(*V*_*bias*_) curve is plotted in Fig. 8a. At zero bias, the measured capacitance is 79.32 fF, which is close to the estimated *C*_0_.

Figure 8a also shows that the capacitance is not constant with a given *V*_*bias*_ sweep. With larger | *V*_*bias*_ |, the capacitor plates move toward each other due to the electrostatic force *F*_*es*_, leading to an increase in capacitance. This change in capacitance induced by *F*_*es*_ provides additional evidence that the proof mass is fully suspended over the substrate. Leveraging these data, the spring constant can be extracted. For each bias point, the equilibrium of forces is assumed to be applicable to the suspended proof mass, where *F*_*es*_ is equal to the force induced by the deformation on the spring (*F*_*spring*_):

4 $${F}_{{es}}={F}_{{spring}}$$

and,

5 $$\begin{array}{l}\qquad\,\,{F}_{{es}}=\frac{{Q}^{2}}{2\varepsilon {N}_{f}{L}_{f}h}\\ \quad{F}_{{spring}}=2k\cdot \Delta d({V}_{{bias}})\end{array}$$

where *Q* is the amount of charge on the capacitor, which equals *C*(*V*_*bias*_)·*V*_*bias*_,

∆*d*(*V*_*bias*_) is the displacement of the proof mass due to *F*_*es*_ at different *V*_*bias*_;

6 $$\Delta d\left({V}_{{bias}}\right)=\frac{\varepsilon {N}_{f}{L}_{f}h}{{C}_{0}}-\frac{\varepsilon {N}_{f}{L}_{f}h}{C\left({V}_{{bias}}\right)}$$

Combining Eqs. (4), (5), and (6), *k* is expressed as follows:

7 $$k=\frac{{[C\left({V}_{{bias}}\right){V}_{{bias}}]}^{2}}{4{\varepsilon }^{2}{N}_{f}^{2}{L}_{f}^{2}{h}^{2}[\frac{1}{{C}_{0}}-\frac{1}{C({V}_{{bias}})}]}$$

With Eq. (7), the spring constant can be extracted from the experimental *C*(*V*_*bias*_) curve. As plotted in Fig. 8a, the extracted *k* is approximately 8.64 N/m, which is approximately half of the estimated value. This result indicates that the SiC-CNT composite has a lower Young’s modulus than that of the bulk SiC. According to Eq. (3), Young’s modulus of the composite coated with 90 nm SiC can be extracted and is 169.61 GPa. This is 3 orders of magnitude improvement over the uncoated CNT array^32^. Compared to ref. ^21^, it can also be concluded that the Young’s modulus of the composite can be increased by depositing more filler material. In separate work, we recently demonstrated that the mechanical properties of the composite do not degrade up to at least 900 °C^33^.

The rest capacitance was also measured at elevated temperatures to investigate the high-temperature compatibility of the nanocomposite. In situ capacitive measurements were conducted from room temperature to 200 °C. The result is shown in Fig. 8b. We can see that the capacitance increases with temperature. An explanation for this effect may be that the fingers expand with respect to temperature, inducing an increasing overlapping area as well as a reduced gap of the capacitor.

**Fig. 9:**
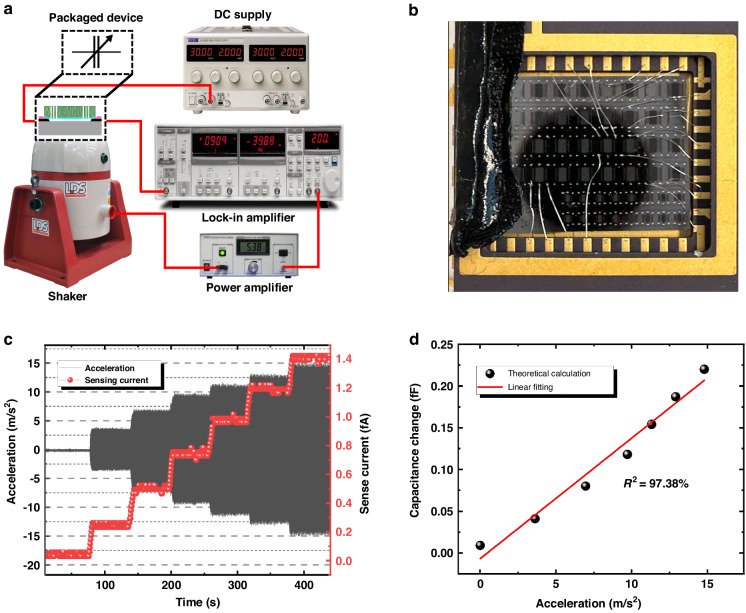
An overview of the measurement setup and results. **a** A schematic overview of the measurement setup for the vibration test. **b** The packaged chip on the dual inline package. **c** Real-time measured acceleration applied to the device and the sensed current (*I*_*sense*_). **d** Derived capacitance change with respect to the acceleration

The accelerometer was also measured on a shaker to verify its response to the vibrating environment. A schematic overview of the corresponding setup is shown in Fig. 9a. The device was packaged in a dual inline package and mounted on a shaker, as shown in Fig. 9b. The shaker was driven by a sinusoidal wave at a certain frequency (*f* = 90 Hz). By tuning the amplitude of the excitation signal, different accelerations can be applied to the device. The acceleration profile was recorded in real time by a commercial accelerometer, as shown in Fig. 9c, where the amplitude of the acceleration (*a*_*max*_) increases from 0 to 14.8 m/s^2^ by steps.

The capacitance change induced by the acceleration is extremely small due to the low sensitivity (estimated to be 0.07 fF/g). Therefore, direct measurement is difficult, and the acceleration versus capacitance change is indirectly measured. As the accelerometer’s movement on the shaker can be approximated as simple harmonic motion, the acceleration can be considered sinusoidal. According to Eq. (1), the capacitance of the sensor is also a sine curve with an amplitude of ∆*C*(*a*_*max*_). The accelerometer was biased at 10 V (*V*_*DC*_) by a constant voltage supply. While shaking, the capacitance changes induce a current, which is sensed by a lock-in amplifier (LIA). The sensed current (*I*_*sense*_) was recorded at the same time as the acceleration and is plotted in Fig. 9c. The measured current demonstrates distinguishable output levels as well as a fast response with respect to different applied accelerations. Within half a period (*T/*2), we define:

8 $${I}_{{sense}}\cdot \frac{T}{2}={V}_{{DC}}\cdot {\int }_{0}^{\frac{T}{2}}\Delta C\left({a}_{\max }\right)\cdot \sin \left(2\pi {ft}\right){{\rm{d}}t}$$

From this equation, the change in capacitance as a function of acceleration can be calculated; thus, the sensitivity can be approximated by linear fitting. The calculated result is plotted in Fig. 9d. The coefficient of determination (*R*^2^) of the linear fit is 97.38%, and the slope of the linear fit curve reveals that the accelerometer sensitivity is approximately 0.14 fF/g. The measured sensitivity is 2 times greater than the theoretical value. This discrepancy can be explained by the fact that the spring is less stiff than predicted, which results in a higher sensitivity of the sensor. Although the current sensitivity of the device is relatively low compared to that of mature Si-based devices, it can still be improved by geometric optimization, such as increasing the rest capacitance, and fabricating springs with less stiffness.

## Conclusion

In this work, we utilized a VACNT array coated with LPCVD a-SiC to produce a SiC-CNT composite, aiming to overcome the bottleneck in fabricating HAR SiC structures. Due to the fast growth rate and vertical growth property of CNTs, the prepatterned CNT array provides an excellent template for fabricating HAR microstructures. By taking advantage of the porous nature of CNT bundles, a-SiC filler can easily penetrate the CNT template and be uniformly coated on each fiber, thus densifying the structure. The electrical resistivity of the composite and the contact properties were studied by the ELM and TLM structures. The conductivity of the SiC-CNT composite was 10^7^ times greater than that of the a-SiC filler material. The TCR of the composite is approximately −315 ppm/K, which is similar to that of pure CNTs. The contact resistivity of the SiC-CNT/TiN interface is 4.63 × 10^*−*4^ Ω cm^2^.

A surface micromachined accelerometer was designed and fabricated to demonstrate using the composite in MEMS applications. The nominal capacitance aligns with the design value, and the *C*-*V* curve indicates that the device was successfully suspended. The Young’s modulus of the composite is extracted from the measured spring constant, which is 169.61 GPa. The high-temperature test showed that the composite has the potential to be applied in harsh environments. The measured sensitivity of the accelerometer is 0.14 fF/g (based on an indirect measurement). Continued work on the mechanical characterization of the composite, real-time measurement of the capacitive output, and integration with ASIC are planned.

### Supplementary information

Fig. A1

Fig. A2

Table A1
