# Supplementary figures and images for: A high aspect ratio surface micromachined accelerometer based on a SiC-CNT composite material
Source: Microsyst Nanoeng. 2024 Mar 22;10:42. doi: 10.1038/s41378-024-00672-x (PMC10957932; doi:10.1038/s41378-024-00672-x)

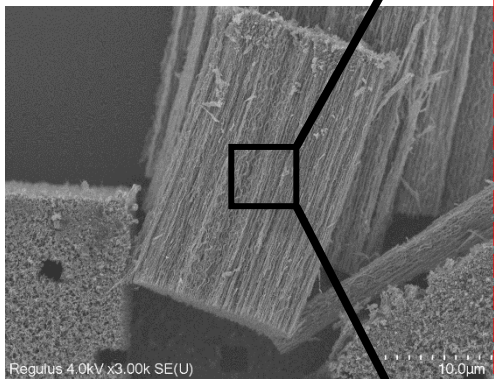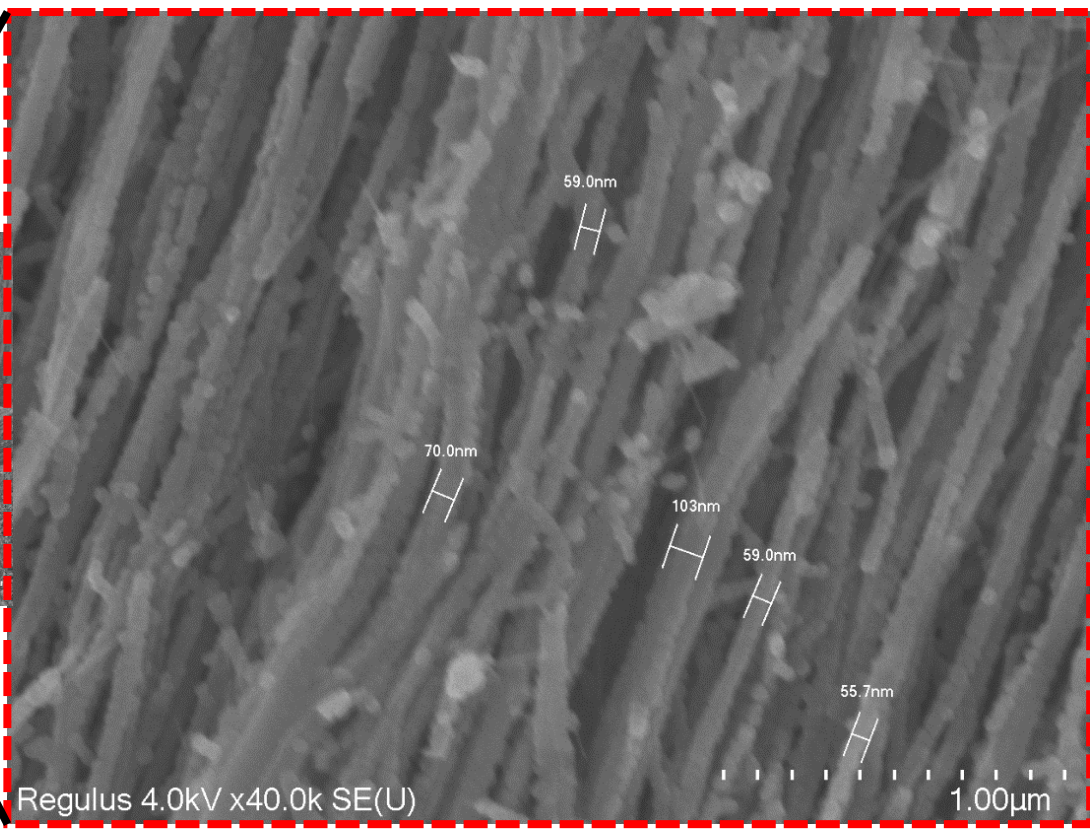

Supplement: Supplementary file 1 — Fig. A1 [file 41378_2024_672_MOESM1_ESM.pdf]

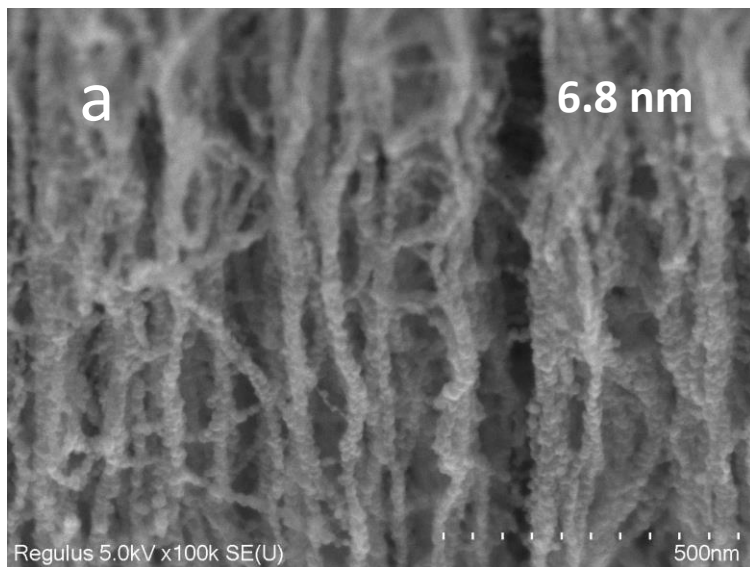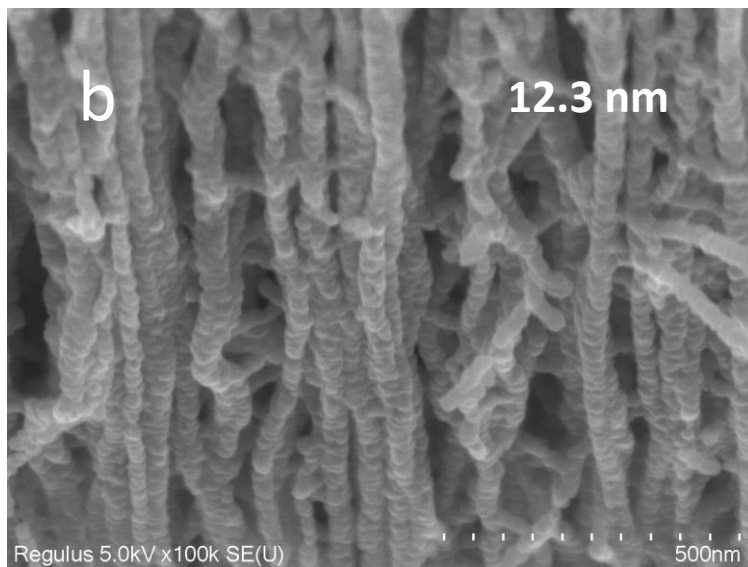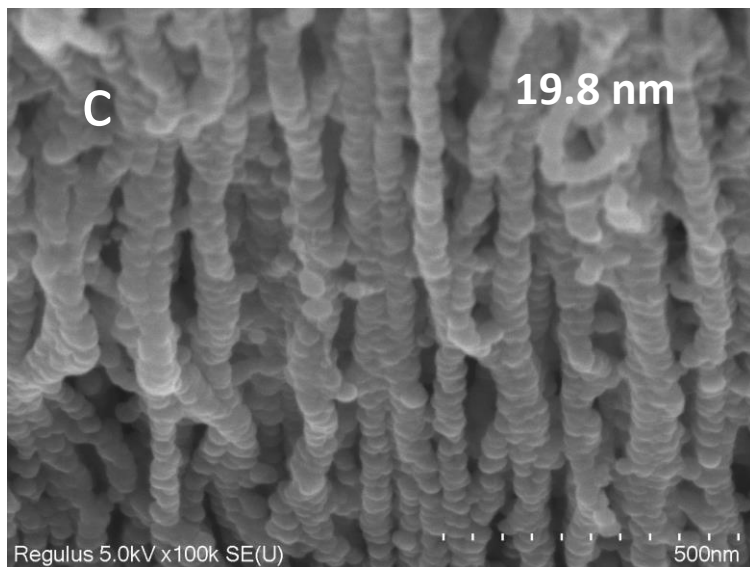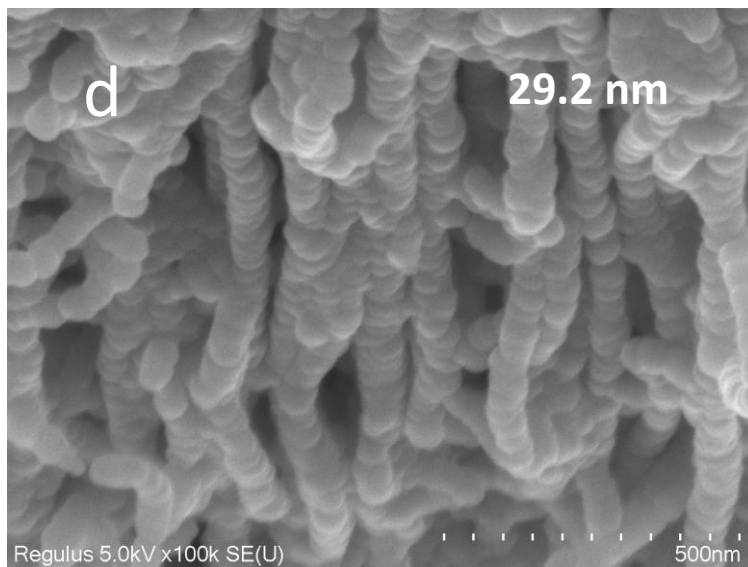

Supplement: Supplementary file 2 — Fig. A2 [file 41378_2024_672_MOESM2_ESM.pdf]
