# Supplementary material for: A high aspect ratio surface micromachined accelerometer based on a SiC-CNT composite material
Source: Microsyst Nanoeng. 2024 Mar 22;10:42. doi: 10.1038/s41378-024-00672-x (PMC10957932; doi:10.1038/s41378-024-00672-x)
Supplement: Supplementary file 3 — Table A1 [file 41378_2024_672_MOESM3_ESM.docx]

# Appendix A Supporting information

**Table A1** The VACNT array height with respect to growth time.

| Growth time (min) | CNT height (*µ*m) |
| --- | --- |
| 1 | 33.65 |
| 2 | 61.09 |
| 3 | 72.26 |
| 5 | 96.30 |

**Fig. A1** SEM image of the cleaved SiC-CNT composite. A zoomed-in view of the cleavage plane is shown in the red box.

**Fig. A2** Zoomed-in view of the VACNT surface after coating with (a) 6.8 nm, (b) 12.3 nm, (c) 19.8 nm, and (d) 29.2 nm a-SiC. With a thicker SiC deposition, a decrease in the void density between individual fibers can be clearly observed.
